# Approach to Higher Wheat Yield in the Huang-Huai Plain: Improving Post-anthesis Productivity to Increase Harvest Index

**DOI:** 10.3389/fpls.2018.01457

**Published:** 2018-10-23

**Authors:** Jianzhao Duan, Yapeng Wu, Yi Zhou, Xingxu Ren, Yunhui Shao, Wei Feng, Yunji Zhu, Li He, Tiancai Guo

**Affiliations:** ^1^State Key Laboratory of Wheat and Maize Crop Science, Collaborative Innovation Center of Henan Grain Crops, Henan Agricultural University, Zhengzhou, China; ^2^Wheat Research Center, Henan Academy of Agricultural Sciences, Zhengzhou, China

**Keywords:** winter wheat, increased yield, harvest index, dry matter, leaf area index, radiation interception

## Abstract

Both increased harvest index (HI) and increased dry matter (DM) are beneficial to yield; however, little is known about the priority of each under different yield levels. This paper aims to determine whether HI or DM is more important and identify the physiological attributes that act as indicators of increased yield. Two field experiments involving different cultivation patterns and water-nitrogen modes, respectively, were carried out from 2013 to 2016 in Huang-Huai Plain, China. Plant DM, leaf area index (LAI), and radiation interception (RI) were measured. Increased yield under low yield levels <7500 kg ha^-1^ was attributed to an increase in both total DM and HI, while increases under higher yield levels >7500 kg ha^-1^ were largely dependent on an increase in HI. Under high yield levels, HI showed a significant negative correlation with total DM and a parabolic relationship with net accumulation of DM during filling. Higher net accumulation of DM during filling helped slow down the decrease in HI, thereby maintaining a high value. Moreover, net DM accumulation during filling was positively correlated with yield, while post-anthesis accumulation showed a significant linear relationship with leaf area potential (LAP, *R*^2^ = 0.404–0.526) and radiation interception potential (RIP, *R*^2^ = 0.452–0.576) during grain filling. These findings suggest that the increase in LAP and RIP caused an increase in net DM accumulation after anthesis. Under DM levels >13,000 kg ha^-1^ at anthesis, maintaining higher LAI and RI in lower layers during grain formation contributed to higher yield. Furthermore, the ratio of upper- to lower-layer RI showed a second-order curve with yield during filling, with an increase in the optimal range with grain development. Pre-anthesis translocation amount, translocation ratios and contribution ratios also showed second-order curves under high yield levels, with optimal values of 3000–4500 kg ha^-1^, 25–35, and 30–50%, respectively. These results confirm the importance of HI in improving the yield, thereby providing a theoretical basis for wheat production in the Huang-Huai Plain.

## Introduction

With the increasing world population, which is estimated to reach 9.4 billion by 2050 (Foulkes et al., 2009), global agriculture in the 21st century is facing a significant increase in crop production. Wheat is one of the most important major food sources providing more than 20% of human calorie consumption (Merchukovnat et al., 2016). Increases in wheat yield are therefore important. Since the 1960s, it has been suggested that increases in the harvest index (HI; the proportion of total dry matter (DM) allocated to harvested grains) result in an increase in grain yield (Nass, 1980; Slafer et al., 1994; Qin et al., 2013). Thus, with the development of wheat breeding, the mean HI of wheat has experienced an increase from 0.35 in 1951–1955 to 0.5 in 1995–2013 (Evans and Fischer, 1999; Foulkes et al., 2009; Wiesmeier et al., 2014), while Austin (1980) predicted that the HI of modern winter wheat could reach a maximum of 0.62. Therefore, since the HI of modern cultivars is already high, it has been suggested that further improvements in yield will rely more on increases in total DM (Aranjuelo et al., 2013; Parry and Hawkesford, 2010; Parry et al., 2010). However, in recent years, breeding of high-yielding wheat has also resulted in an increase in total DM to >18,000 kg ha^-1^ at maturity. Some studies suggest, therefore, that further increases in wheat yield are now largely dependent on increases in HI, rather than increases in DM (Aranjuelo et al., 2013; Fan et al., 2017). Moreover, agronomic practices such as nitrogen application and irrigation technology have further aided development, leading some to suggest that wheat yield has reached a plateau (Qin et al., 2015; Yu et al., 2017). Nevertheless, increased food production remains a priority for future world populations. In wheat production, both a high HI and DM are beneficial to increased yield; however, the contribution of each differs in different regions. Understanding the respective relationships between yield, DM, and HI in modern cultivars is therefore necessary, particularly under different yield levels, as is confirming the respective importance of DM and HI in terms of sustainable high yield.

Grain yield is the product of both DM and HI, with increases in yield resulting from this co-operative relationship. However, the contributions of DM and HI to yield in different regions and under different productivity levels are likely different. For a specific region, where light and temperature resources are stable and DM production potential is basically constant, increasing HI is a feasible approach to improving yield. However, in reality, DM production tends to reach saturation under high yield levels, and increased yield becomes more dependent on increases in HI. Research further suggests that improvements in HI require balance between DM production before and after anthesis (Fischer, 1981), with overproduction prior to anthesis and underproduction afterward resulting in DM accumulation in the stems and leaves rather than the grains, thereby decreasing the HI. Prior to anthesis, photosynthates are mainly stored in vegetative organs, after which they are transported to reproductive organs (Papakosta and Gagianas, 1991; Zhang et al., 2008). Previous studies suggest that saturation of pre-anthesis DM is important for growing spikes and the grain number per spike, resulting in a large sink (Fischer, 1985; Slafer et al., 2014). Thus, post-anthesis photosynthetic capacity and DM mobilization from vegetative organs to grains therefore determines final grain filling. Accordingly, post-anthesis DM production and mobilization, and translocation of pre-anthesis assimilates have therefore been investigated (Fischer, 1981; Papakosta and Gagianas, 1991; Ercoli et al., 2008; Zhang et al., 2008, 2016). In general, the nutrients required for grain filling are obtained from assimilation of photosynthetic products and remobilization of stem and leaf DM. Although the degree of remobilization from vegetative organs is conducive to HI, and therefore yield, it has limitations. The more photosynthates produced after anthesis, the more that are transported to the grains and the higher the grain yield under higher yield levels. Furthermore, while studies have examined the translocation and contribution of vegetative organ DM under different ecological regions and production conditions (Papakosta and Gagianas, 1991; Ercoli et al., 2008), little is known about the optimal translocation and contribution ratios under different yield levels. Plant DM production requires interception and absorption of light radiation for photosynthesis, with the interception of radiation thought to be correlated with the rate of photosynthesis (Richards, 2000). Thus, with wheat grain filling, green leaves gradually undergo senescence, decreasing the capacity for interception of radiation, and therefore, photosynthesis. Previous studies further suggest that a higher amount and duration of light interception during grain filling is conducive to increased photosynthesis and post-anthesis DM production (Richards, 2000; Schierenbeck et al., 2016). However, despite these findings, few studies have examined the effect of leaf area index (LAI) and intercepted radiation under different spatial altitudes on DM production under different DM levels.

In recent years, an increasing number of modern high-yielding wheat cultivars have been bred and popularized. To help understand how to achieve a high HI and yield via an optimal canopy structure and radiation interception, we carried out a series of experiments in the Huang-Huai Plain, which accounts for approximately 55% of the wheat planting area and 60% of wheat production in China. The aims were to: (I) describe the main factors affecting increased grain yield under different production levels (> and <7500 kg ha^-1^); (II) identify the optimal translocation and contribution ratios of pre-anthesis vegetative organ DM; (III) clarify the spatial characteristics of canopy LAI and intercepted radiation during grain filling; and (IV) characterize the physiological indicators of increased yield. The results provide a theoretical basis for further increases in grain yield in the Huang-Huai Plain.

## Materials and Methods

### Experimental Sites

Two field experiments were carried out at three sites in the Huang-Huai Plain, China, from October 2013 to June 2016 – Shangshui (114° 29′ E, 33° 32′ N), Kaifeng (114° 37′ E, 34° 44′ N), and Wenxian (112° 99′ E, 34° 92′ N), in southern, central, and northern Henan Province, respectively. The climatic conditions of the study regions result in a long tillering stage (110–120 days), young ear differentiation period (160–170 days), and short grain filling stage (35–40 days), all of which benefit the grain number per unit area but not grain filling. Specific properties of the plow layer soil are shown in Table 1. In all three experimental sites, the preceding crop was maize, with the straw mechanically returned to the field after harvest.

**Table 1:** Basic physicochemical properties of the field experiment sites at a soil depth of 0–40 cm.

Items^∗^	Shangshui			Kaifeng			Wenxian		
	2013–2014	2014–2015	2015–2016	2013–2014	2014–2015	2015–2016	2013–2014	2014–2015	2015–2016
pH	7.81	8.07	7.92	8.13	8.39	8.15	8.27	8.40	8.29
TN	0.95	1.25	1.33	0.80	0.96	1.01	0.89	0.93	0.95
AEN	90.35	110.00	118.33	74.70	85.43	99.25	93.90	100.54	102.95
OM	16.68	18.47	18.78	12.96	14.86	15.12	12.57	13.93	14.02
APP	12.94	27.28	15.70	12.72	13.13	11.56	12.56	16.36	15.13
APS	116.88	124.67	119.30	136.26	164.25	157.75	190.59	191.50	176.59
Texture	LCBS	LCBS	LCBS	Clay	Clay	Clay	FACS	FACS	FACS

*^∗^TN, total N (g kg^-1^); AEN, alkaline extractable N (mg kg^-1^); OM, organic matter (g kg^-1^); APP, available phosphorus (mg kg^-1^); APS, available potassium (mg kg^-1^); LCBS, lime concretion black soil; FACS, fluvo-aquic clay soil.*

With improvements in breeding and cultivation management technology, wheat yield in China has significantly improved, especially in northern wheat-growing areas, from 3000 kg ha^-1^ in the 1950s to 5500 kg ha^-1^ in the 1980s and to more than 7500 kg ha^-1^ since 2010 (Wei et al., 2013; Ru et al., 2015; Wang H.G. et al., 2015). Recent high yield standards are therefore classified as 7500 kg ha^-1^ in large areas of wheat production, with the production characteristics at a yield of >7500 kg ha^-1^ differing from those at <7500 kg ha^-1^. As a result, the approaches required to increase grain yield differ between these two yield levels. In this study, we therefore chose to use these two yield levels as the cut off between high and low yield production.

### Experimental Design

#### Experiment 1. Cultivation Patterns

Four cultivation patterns were tested using the wheat cultivar Yumai 49–198: local farming practices (FP); optimized cultivation compared with FP (OFP); super high-yielding cultivation (SH); and high-yielding high-efficiency cultivation (HH). In all cases and in all sites, the date of sowing was Oct 12–17. Under FP, rotary tillage (to about 15 cm) was carried out before sowing with no rolling after seeding. Irrigation was carried out after sowing and during the returning green stage. Equal row spacing of 20 cm and a large sowing quantity (225 kg ha^-1^) were applied. Under OFP, SH, and HH, mechanical deep plowing (more than 25 cm) was adopted, with multiple rotary harrowing and rolling after seeding. Irrigation was carried out after sowing and during jointing. Equal row spacing of 20 cm was adopted in OFP, and 17 cm in SH and HH. A sowing quantity of 150 kg ha^-1^ was applied in OFP, and 120 kg ha^-1^ in both SH and HH. Irrigation was carried out, and organic, microelement, and phosphate and potassium fertilizers were applied before sowing (Table 2). Under FP, all N (urea), phosphate (P: P_2_O_5_), and potassium (K: K_2_O) fertilizers were applied as base fertilizer before sowing. Under OFP and SH, 50% of the N fertilizer and all P and K fertilizers were applied before sowing, with the remaining 50% N fertilizer applied at jointing. Under HH, 40% of the N fertilizer and 70% of the P and K fertilizers were applied before sowing, with the remaining 60 and 30%, respectively, applied at jointing. All organic (1.63% N, 1.54% P_2_O_5_, and 0.85% K_2_O) and microelement fertilizers (ZnSO_4_) were applied before sowing. Details of the fertilizer and irrigation regimes are shown in Table 2.

**Table 2:** Irrigation and fertilization management under each cultivation pattern in the field experiments.

Treatment	Rate of fertilizer application (kg ha^-1^)					Irrigation amount at each stage (m^3^ ha^-1^)			
^∗^	N	P_2_O_5_	K_2_O	ZnSO_4_	Organic fertilizer	Soil moisture water	Turning green stage	Jointing stage	Blossom filling stage
FP	225	75	60	0	0	900	600	0	900
OFP	180	75	60	0	0	600	0	900	750
SH	300	150	150	15	1500	600	0	900	900
HH	240	90	90	15	1500	600	0	900	750

*^∗^FP, local farming practice; OFP, optimized cultivation compared with FP; SH, super high-yielding cultivation; HH, high-yielding and high-efficiency cultivation.*

The four cultivation patterns therefore involved individual characteristics. Specifically, FP treatment involved a larger seeding rate, single basal application of fertilizers before sowing, and irrigation during the returning green stage. OFP treatment focused on a reduced fertilizer application rate, with proper top-dressing and irrigation at jointing compared to FP. SH treatment was carried out to achieve the highest possible yield regardless of the cost of fertilization and irrigation, while HH treatment was focused on achieving high yield and high efficiency by reducing the fertilizer application rate and optimizing nitrogen application (i.e., top dressing) and irrigation compared to SH. This treatment followed a randomized block design with three replications. Each plot was 165 m^2^ (15 m in length and 11 m wide).

#### Experiment 2. Water-Nitrogen Modes

Water-nitrogen mode experiments were carried out using the wheat cultivar Aikang 58, sown on Oct 12–17 in Shangshui and Wenxian. In Shangshui, the experiments consisted of a combination of two irrigation regimes (no irrigation and single irrigation of 750 m^3^ ha^-1^ at jointing) and four N rates (0,180, 240, and 300 N kg ha^-1^). In Wenxian, the experiments consisted of three irrigation regimes (no irrigation, single irrigation of 750 m^3^ ha^-1^ at jointing, and irrigation of 750 m^3^ ha^-1^ at jointing plus anthesis) and four N rates (as listed above). As a base fertilizer, 50% N fertilizer (Wenxian) or 70% N fertilizer (Shangshui) was applied along with all P (100 kg P ha^-1^) and K (150 kg K ha^-1^) fertilizers before sowing. The remaining N fertilizer was applied at jointing. Experimental plots were arranged in a split-plot design with three replicates, with each plot measuring 40.6 m^2^ (7 m in length and 5.8 m wide). The main plot was assigned to the irrigation regime and the subplot to the N rate.

### Growing Season Conditions and Crop Phenology

**FIGURE 1:**
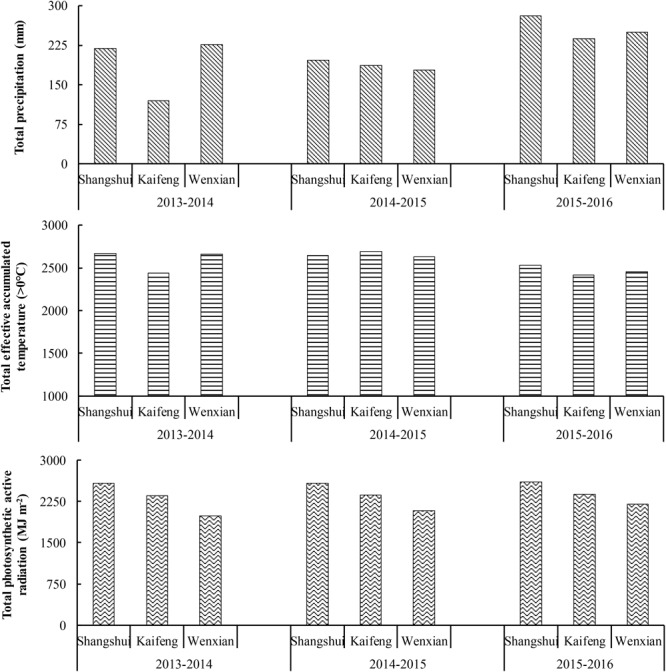
Meteorological data during each growing season.

The experimental sites have a warm temperate, semi-humid, continental monsoon climate. Detailed climatic conditions of each site in each year are shown in Figure 1. In general, the total rainfall, effective accumulated temperature (>0°C), and photosynthetic active radiation were higher in Shangshui than in Kaifeng and Wenxian, while photosynthetic active radiation was higher in Kaifeng than in Wenxian. From 2013 to 2016, the total ranges in effective accumulated temperature (>0°C) and photosynthetic active radiation varied little in Shangshui. However, in all the three sites, rainfall varied between 2015 and 2016, and was higher than in previous years, thereby having an advantageous effect on wheat growth.

#### Sampling and Measurements

##### Biomass

Timing of each developmental stage from anthesis to maturity was determined as the point when 50% of plants reached a specific standard. Aboveground DM was measured at different growth stages (anthesis, early grain filling, mid-grain filling, and maturity). Twenty plants were randomly sampled three times from anthesis to maturity, resulting in a total of 60 plants and covering an area approximating 0.2 m^2^. They were divided into stems, leaves, and spikes, oven dried at 65°C, and then used to determine DM. Net DM accumulation during filling was calculated as the difference between the total DM at anthesis and maturity.

##### Leaf area index (LAI) and radiation interception (RI)

Green leaf area can be represented by the LAI, which is further divided according to spatial architecture into upper- and lower-layer LAI. The total LAI (LAIt) was determined at the base of the plants using a plant canopy analyzer (LAI 2200C, LI-COR, Lincoln, NE, United States), while the upper-layer LAI (LAIu) was determined at the third leaf from the top of the flag. Lower-layer LAI (LAIL) was then calculated by the difference between LAIu and LAIt. Sampling was carried out at anthesis, early grain filling (7 days after anthesis), mid-grain filling (15 days after anthesis), and 21 days after anthesis.

The ability to intercept radiation was estimated by measuring the photosynthetic active radiation (PAR) from 11:00 to 13:00 on a sunny day under the canopy (MJ m^-2^) using a 1 m long linear sensor (LI 191S, LI-COR Inc., Lincoln, NE, United States). Incident PAR (Io) and reflected PAR (R) were measured at the top of the canopy, and mid and base incident PAR were measured at the same positions as used to determine LAI, i.e., the third leaf from the top of the flag (I_m_) and the base plant (I_b_). The ratio of upper- to lower-layer radiation interception (f) was then calculated as follows:

$$\begin{array}{c} f=(I_{0}-I_{m}-R)/(I_{m}-I_{b}) \end{array}$$

and the radiation interception rate (F) defined as the ratio of total radiation interception to incident PAR (I_o_) was calculated as follows:

$$\begin{array}{c} F=(I_{0}-I_{b}-R)/I_{0} \end{array}$$

The accumulation of leaf area and RI with time (days) has a significant effect on wheat production; therefore, the average leaf area potential (LAP) and radiation interception potential (RIP) were calculated as follows:

$$\begin{array}{c} \mathrm{LAP}=1/2{\left({\mathrm{LAI}}_{t1}+{\mathrm{LAI}}_{t2}\right)}^{*}T \end{array}$$

where LAI_t1_ and LAI_t2_ represent two adjacent LAI values, and T is the number of days between measurements.

$$\begin{array}{c} \mathrm{RIP}=1/2{\left(F1+F2\right)}^{*}T \end{array}$$

where F1 and F2 represent two adjacent radiation interception rate (F) values, and T is the number of days between measurements.

##### Grain yield and HI

At maturity, an area of 5 m^2^ was harvested, and the harvested samples were threshed and dried to determine the grain yield (kg ha^-1^). HI was determined as the ratio of grain yield to total DM at maturity.

### Statistical Analysis

Yield, DM and HI data were obtained from the cultivation and water-nitrogen mode experiments carried out from 2013 to 2016 (total: 288 samples). LAI and light radiation data were obtained from the cultivation experiments carried out in Shangshui and Kaifeng from 2014 to 2016 and the water-nitrogen mode experiments carried out in Shangshui and Wenxian from 2014 to 2016 (total: 168 samples). Experimental data were analyzed and processed using Microsoft Office 2013. Variance analysis was carried out using Duncan’s multiple range test at a significance level of 5% and Pearson’s correlation analysis at *P* < 0.05 using SPSS version 19.0.

## Results

### Relationships Between Yield, DM and HI

As shown in Table 3, the cultivation pattern experiments involving Yumai 49–198 showed that SH and HH resulted in higher yield, GN (grain number per unit area), HI, and DM than FP treatment in all the three locations (Table 4). Yield, GN, and DM showed the same trend between locations: Shangshui > Wenxian > Kaifeng, while the thousand grain weight (TGW), HI, and total DM revealed variations. Meanwhile, the water-nitrogen mode experiments using Aikang 58 had a significant effect on yield, the yield components, HI, and DM in Shangshui and Wenxian (Table 5). Yield, GN, HI, and DM increased with increasing N application rate from 0 to 240 kg ha^-1^ and increasing irrigation application from 0 to 2 times. Under high N application rates, the above indicators showed no significant differences between N240 and N300, decreasing under W2. Overall, therefore, irrigation, nitrogen, and cultivation management strongly affected yield and other physiological indicators.

**Table 3:** Descriptive statistics of yield, yield components, HI, and DM accumulation.

Component^∗^	*N*	Average	Maximum	Minimum	*SD*
Yield	288	7222.84	10194.67	2980.08	1715.46
GN	288	18.96	32.17	6.18	5.57
TGW	288	46.64	54.98	37.84	3.58
HI	288	0.42	0.53	0.27	0.05
DM_A_	288	12733.74	18268.22	5884.80	2796.04
DM_M_	288	17280.67	23384.75	9038.83	3502.11
DM_AM_	288	4480.50	6877.28	2222.52	1096.22
TDM	288	2675.90	5185.75	185.79	1148.60

*^∗^GN, grain number per unit area (10^*3*^⋅m^-*2*^); TGW, thousand grain weight (g); HI, harvest index; DM_*A*_, dry matter at anthesis (kg ha^-*1*^); DM_*M*_, dry matter at maturity (kg ha^-*1*^); DM_*AM*_, dry matter accumulation from anthesis to maturity (kg ha^-*1*^); TDM, dry matter translocation amount after anthesis (kg ha^-*1*^).*

**Table 4:** ANOVA of grain yield, yield components, HI, and DM accumulation under each cultivation pattern experiment (cultivar Yumai 49–198).

Locations	Treatments	Yield	GN	TGW	HI	DM_A_	DM_M_	DM_AM_	TDM
Shangshu	FP	7298.27b	18.86b	48.73a	0.40b	13542.50b	18281.85b	4550.48b	2558.93b
i	JH	8007.41b	21.99ab	45.90ab	0.44a	13944.50ab	18523.34b	4578.85b	3428.57a
	SH	9313.61a	25.52a	44.98b	0.44a	15364.96a	21392.65a	6027.78a	3285.91ab
	HH	9081.40a	25.17a	45.25b	0.43ab	15572.77a	21295.46a	5744.93a	3358.71ab
Kaifeng	FP	6922.99b	18.34b	46.13a	0.42b	11998.81b	16439.48b	4440.68b	2482.32b
	JH	7523.00b	20.63ab	46.84a	0.42b	13983.95ab	18297.46ab	4424.69b	3209.49ab
	SH	8819.50a	23.23a	45.46a	0.46a	14425.34a	19253.04a	5016.59ab	3991.81a
	HH	8644.10a	23.83a	43.77a	0.44ab	14275.03ab	19763.25a	5510.42a	3155.88ab
Wenxian	FP	7139.25c	18.25b	47.46a	0.42b	12727.80b	17168.56b	4140.72b	2698.48b
	JH	7830.30b	21.35a	48.18a	0.42b	14178.54ab	18517.02ab	4338.48ab	3491.82a
	SH	9081.49a	24.13a	45.74a	0.46a	14664.02a	19797.38a	4955.62ab	3948.13a
	HH	8762.29a	23.87a	45.79a	0.45a	14454.75ab	19545.89a	5091.15a	3671.15a

*FP, local farming practice; OFP, optimized cultivation compared with FP; SH, super high-yielding cultivation; HH, high-yielding and high-efficiency cultivation; GN, grain number per unit area (10^*3*^⋅m^-*2*^); TGW, thousand grain weight (g); HI, harvest index; DM_*A*_, dry matter at anthesis (kg ha^-*1*^); DM_*M*_, dry matter at maturity (kg ha^-*1*^); DM_*AM*_, dry matter accumulation from anthesis to maturity (kg ha^-*1*^); TDM, dry matter translocation amount after anthesis (kg ha^-*1*^).*

**Table 5:** ANOVA of grain yield, yield components, HI, and DM accumulation under each water-nitrogen mode experiment (cultivar Aikang 58).

Location	Treatment^∗^	Yield	GN	TGW	HI	DM_A_	DM_M_	DM_AM_	TDM
Shangshui	W0N0	3956.77e	9.49d	48.23ab	0.34b	8616.36c	11684.75d	3068.39d	888.38e
	W0N180	5733.47c	14.51bc	46.52ab	0.43a	9853.93bc	13425.55cd	3671.58cd	2161.84cd
	W0N240	5890.90c	14.29bc	46.71ab	0.40a	10825.34b	14651.13c	3792.50bcd	2065.11cd
	W0N300	6453.04c	15.69b	49.01a	0.43a	11156.87b	15238.30c	4081.43bc	2371.61bc
	W1N0	4954.68d	12.67c	49.84a	0.39a	9117.81c	12638.72d	3520.90cd	1433.77de
	W1N180	7283.04b	19.57a	47.23ab	0.41a	13146.15a	17727.23b	4100.26bc	2701.97abc
	W1N240	8076.29a	21.66a	44.86b	0.43a	14202.48a	18908.55ab	4556.94ab	3370.22a
	W1N300	8126.02a	20.98a	46.40ab	0.42a	14520.23a	19607.21a	5031.49a	3039.05ab
Wenxian	W0N0	3758.05e	8.19d	50.92a	0.34b	8178.96e	11172.37e	2993.41g	764.6d
	W0N180	6506.46bc	15.75b	45.58c	0.42a	10686.75cd	15345.41bc	4658.66bcd	1847.80c
	W0N240	6233.01c	16.77b	46.81bc	0.42a	10841.07cd	15058.80bc	4217.73cde	2015.28bc
	W0N300	6541.63bc	17.33b	46.11bc	0.43a	11482.79cd	15246.02bc	3871.63def	2778.40ab
	W1N0	4493.83de	11.20cd	49.61ab	0.35b	9841.63de	12896.00de	3087.72fg	1439.46cd
	W1N180	7311.11b	21.20a	44.51c	0.43a	12499.05bc	17072.83b	4573.78bcde	2737.33ab
	W1N240	8456.60a	20.87a	45.83c	0.42a	14586.38a	20255.81a	5024.96abc	2787.16ab
	W1N300	8319.03a	20.81a	46.51bc	0.42a	14748.87a	19809.18a	4714.66bcd	3258.72a
	W2N0	5222.99d	13.92bc	47.87abc	0.38ab	9996.08de	13744.44cd	3748.36efg	1474.63cd
	W2N180	8247.55a	20.58a	45.23c	0.43a	14249.06ab	19451.20a	4913.26abc	3045.41a
	W2N240	8653.38a	22.74a	46.12bc	0.42a	15121.30a	20456.89a	5291.18ab	3317.79a
	W2N300	8489.27a	23.37a	44.48c	0.42a	14675.56a	20315.70a	5636.83a	2849.12ab

*^∗^Experimental treatments consisted of: no irrigation under four N rates (0,180, 240, and 300 N kg ha^-*1*^; W0N0, W0N180, W0N240, and W0N300); single irrigation at jointing under four N rates (W1N0, W1N180, W1N240, and W1N300); and irrigation at jointing plus anthesis under four N rates (W2N0, W2N180, W2N240, and W2N300). GN, grain number per unit area (10^*3*^⋅m^-*2*^); TGW, thousand grain weight (g); HI, harvest index; DM_*A*_, dry matter at anthesis (kg ha^-*1*^); DM_*M*_, dry matter at maturity (kg ha^-*1*^); DM_*AM*_, dry matter accumulation from anthesis to maturity (kg ha^-*1*^); TDM, dry matter translocation amount after anthesis (kg ha^-*1*^).*

**FIGURE 2:**
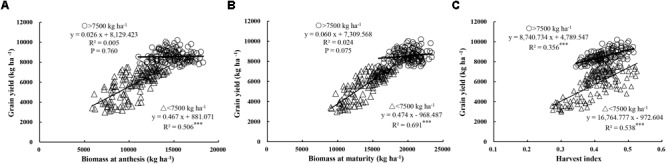
Relationships between grain yield and biomass at anthesis **(A)** and maturity **(B)**, and harvest index **(C)**, respectively. Significant at ^∗^*p* = 0.05, ^∗∗^*p* = 0.01, and ^∗∗∗^*p* = 0.001.

In this study, yield showed a similar relationship with GN, DM, and HI in both cultivars (Yumai 49–198 and Aikang 58), and the experimental data from both experiments in the three locations was therefore pooled for analysis. Figures 2A,B and Table 6 show the relationship between yield and aboveground DM (biomass), yield components, and HI. These relationships showed larger differences at the yield threshold of 7500 kg ha^-1^, 7500 kg ha^-1^ acting as the dividing line for analysis of the contribution of DM to yield. Here, a yield of <7500 kg ha^-1^ was defined as a low yield level, while >7500 kg ha^-1^ was a high yield level. Under low yield levels, significant positive linear relationships were observed between yield and total DM at both anthesis (*R*^2^ = 0.506, *P* < 0.001) and maturity (*R*^2^ = 0.691, *P* < 0.001). Meanwhile, under high yield levels, poor correlations were observed at both stages (*P* > 0.05). Yield was significantly related to HI at both yield levels (Figure 2C; *R*^2^ = 0.356, *P* < 0.001 and *R*^2^ = 0.538, *P* < 0.001 for >7500 kg ha^-1^ and <7500 kg ha^-1^, respectively). These findings suggest that improvements in yield under low yield levels are attributable to increases in both DM and HI, while increases under high yield levels are mainly dependent on marked increases in HI. Furthermore, the total DM under high yield levels tended to be higher than under low yield levels. Moreover, as the total DM continued to increase, yield tended to reach saturation, suggesting that further improvements in yield are dependent on HI.

**Table 6:** Pearson’s correlation between yield and yield components, HI, and DM accumulation (*n* = 288).

	>7500 (kg ha^-1^)		<7500 (kg ha^-1^)		All (kg ha^-1^)	
	GN	TGW	GN	TGW	GN	TGW
Yield	0.55^∗∗^	–0.11	0.84^∗∗^	–0.38^∗∗^	0.88^∗∗^	–0.44^∗∗^
HI	0.39^∗∗^	–0.15	0.54^∗∗^	–0.32^∗∗^	0.50^∗∗^	–0.31^∗∗^

*^∗∗^Significant at 0.01 probability. GN, grain number per unit area (10^*3*^⋅m^-*2*^); TGW, thousand grain weight (g); HI, harvest index; DM_*A*_, dry matter at anthesis (kg ha^-*1*^); DM_*M*_, dry matter at maturity (kg ha^-*1*^); DM_*AM*_, dry matter accumulation from anthesis to maturity (kg ha^-*1*^).*

As shown in Table 6, yield and HI showed a significant positive correlation with GN and a negative correlation with TGW, both at high and low yield levels. These findings suggest that high GN is conducive to high yield. In addition, yield components determine the sink size and affect the HI. The above results suggest that increasing GN contributes to a high HI.

### Effect of Total DM and Net DM Accumulation on HI and Yield

**FIGURE 3:**
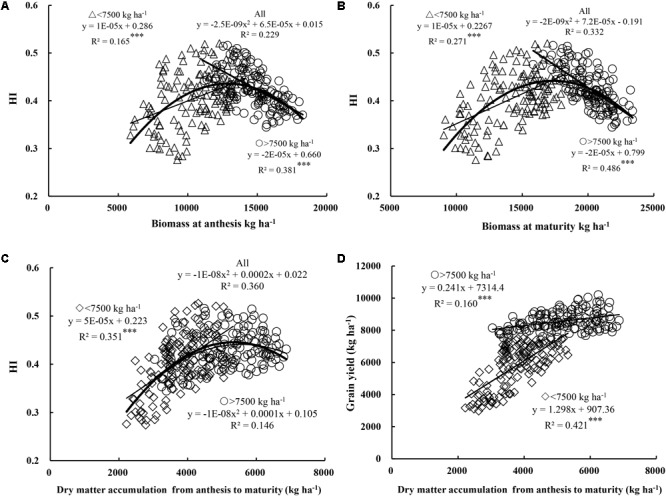
Relationships between harvest index (HI) and biomass at anthesis **(A)** and maturity **(B)**, and relationships between DM accumulation from anthesis to maturity and HI **(C)** and grain yield **(D)**, respectively. Significant at ^∗^*p* = 0.05, ^∗∗^*p* = 0.01, and ^∗∗∗^*p* = 0.001.

HI exhibited a parabolic trend with total DM at both anthesis and maturity when experimental data were pooled (Figures 3A,B). However, the relationship between HI and total DM differed according to yield level. A positive correlation was observed between HI and total DM at anthesis and maturity under low yield levels (*R*^2^ = 0.165–0.271, *P* < 0.001), while, in contrast, a significant negative correlation was observed at anthesis (*R*^2^ = 0.381, *P* < 0.001) and maturity (*R*^2^ = 0.486, *P* < 0.001) under high yield levels. Moreover, as shown in Figures 2A,B, 3A,B, DM of 13,000 kg ha^-1^ at anthesis and 18,000 kg ha^-1^ at maturity were required to determine the dynamic relationships between yield, HI, and DM. In particular, DM accumulation during filling plays an important role in grain filling. When data were pooled, HI showed a parabolic relationship with DM accumulation from anthesis to maturity (Figure 3C). Meanwhile, under low yield levels, HI showed a positive correlation (*R*^2^ = 0.351, *P* < 0.001) with net accumulation of DM during grain filling. These findings suggest that under low yield levels, the net accumulation of DM during filling had a stronger significant effect on HI than total DM at both anthesis and maturity. In contrast, under high yield levels, HI showed a parabolic relationship with net accumulation of DM during grain filling. In other words, excessive total DM caused a decrease in HI, while high net accumulation during grain filling maintained a high HI, thereby helping alleviate the negative correlation between HI and DM at anthesis and maturity. In addition, as shown in Figure 3D, yield exhibited a significant positive correlation with DM accumulation during anthesis to maturity under both yield levels (*R*^2^ = 0.160–0.421, *P* < 0.001). Overall, an increase in net accumulation of DM from anthesis to maturity is therefore beneficial in terms of HI and increased yield.

### Effects of Leaf Area and Incident Radiation on Net DM Accumulation During Filling

**FIGURE 4:**
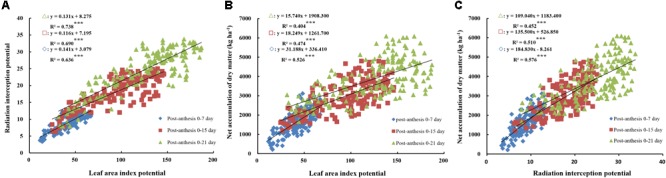
Relationship between radiation interception (RI) potential and leaf area index (LAI) potential **(A)**, and relationships between dry matter (DM) accumulation from anthesis to maturity, radiation interception **(B)** and leaf area index **(C)** potential. Significant at ^∗^*p* = 0.05, ^∗∗^*p* = 0.01, and ^∗∗∗^*p* = 0.001.

Significant positive linear relationships were observed between LAP, RIP, and net DM accumulation at 0–7, 0–15, and 0–21 days after anthesis (Figures 4A–C). Moreover, the three slopes of the regression equations between net DM accumulation and LAP and RIP decreased with the advancement of grain filling, possibly due to gradual leaf senescence and decreased RI. RIP generated a higher determination coefficient compared to LAP when fitted to photosynthetic production, which is relevant in terms of light reflection, transmission, and absorption. As shown in Figure 4A, LAP also showed a significant positive linear relationship with RIP at 0–7, 0–15, and 0–21 days after anthesis (*R*^2^ = 0.636–0.738, *P* < 0.001), suggesting that an increase in LAP from 0 to 21 days after anthesis causes an increase in RIP. A reasonable canopy structure with optimal leaf distribution helps promote the light absorption capacity of different leaf layers, reducing light reflection and the transmission ratio. Thus, the higher the LAP and stronger the RIP, the more photosynthetic products there are to meet the needs of grain filling.

### Respective Relationships Between Grain Yield, RI, and LAI During Grain Filling

**FIGURE 5:**
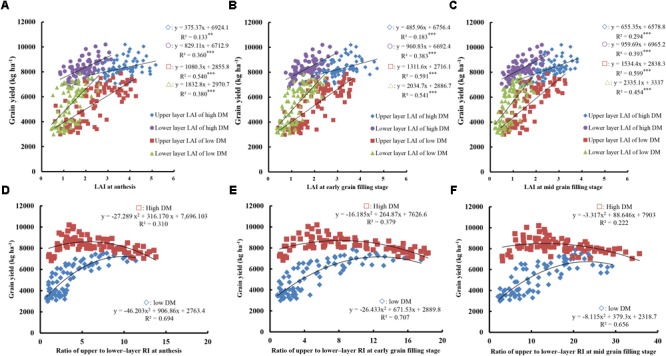
Relationships between grain yield and the ratio of upper- to lower-layer RI, and LAI at anthesis, early, and mid-grain filling. **(A–C)** Relationships between yield and LAI at anthesis **(A)**, early grain filling stage, **(B)** and mid grain filling stage **(C)**. **(D–F)** Relationships between yield and ratio of upper- to lower-layer RI at anthesis **(D)**, early grain filling stage, **(E)** and mid grain filling stage **(F)**. High DM, DM at anthesis >13,000 kg ha^-1^; low DM, DM at anthesis <13,000 kg ha^-1^. Significant at ^∗^*p* = 0.05, ^∗∗^*p* = 0.01, and ^∗∗∗^*p* = 0.001.

Leaf vertical distribution and RI showed significant differences between different total DM levels, with RI directly affected by the spatial distribution of the leaves (Figure 5). Meanwhile, LAI gradually decreased with leaf maturation and yellowing after anthesis. As shown in Figures 5A–C, the relationship between grain yield and LAI was determined by the canopy layer, growth stage, and total DM level, suggesting that maintaining a green leaf area for longer period and slowing down the aging process are conducive to increased yield. LAI was positively correlated with grain yield regardless of the condition of the canopy layer, growth stage, and total DM level, suggesting that high LAI is beneficial in terms of yield. When the total DM at anthesis was <13,000 kg ha^-1^, LAI resulted in a higher *R*^2^ in upper compared to lower layers. Moreover, the *R*^2^ of the upper layer showed a more prominent trend with the advancement of grain filling (*R*^2^ = 0.540 at anthesis, 0.591 at early filling, and 0.599 at mid filling, all *P* < 0.001). However, when the total DM at anthesis was >13,000 kg ha^-1^, LAI demonstrated a higher *R*^2^ in lower compared to upper layers, and the *R*^2^ of lower layers showed a more prominent advantage with grain filling (*R*^2^ = 0.360 at anthesis, 0.383 at early filling, and 0.393 at mid filling, all *P* < 0.001).

As shown in Figures 5D–F, grain yield followed curves of a second-order equation with ratios of upper- to lower-layer RI from anthesis to mid-filling stages under both total DM levels. These findings suggest that the RI ratio has an optimal range in terms of yield. The optimum RI ratio under high DM levels was lower than that under low DM levels at anthesis, early, and mid-grain filling stages. Moreover, the optimum RI ratio gradually increased with the development of grain filling, with respective optimal ranges of 2–6, 3–8, and 7–13 at anthesis, early filling, and mid filling under a high DM level and 6–10, 8–13, and 12–25 under a low DM level, respectively. These findings suggest that increases in lower-layer RI and a relatively low RI ratio are beneficial to yield when DM levels are >13,000 kg ha^-1^. In contrast, higher upper-layer RI and a relatively high RI ratio are conducive to increased yield when DM levels are <13,000 kg ha^-1^.

### Contribution of DM Translocation to Yield and Growth Indictors of High Yield

**FIGURE 6:**
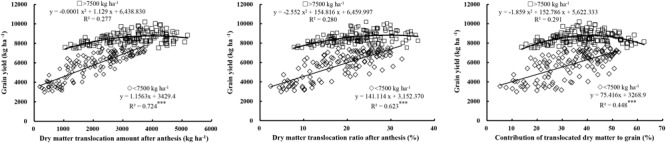
Relationships between grain yield and the translocation amount, translocation ratio and contribution ratio of pre-anthesis dry matter (DM) to grains. Significant at ^∗^*p* = 0.05, ^∗∗^*p* = 0.01, and ^∗∗∗^*p* = 0.001.

Grain yield showed a significant positive linear correlation with the translocation amount (*R*^2^ = 0.724, *P* < 0.001), translocation ratio (*R*^2^ = 0.623, *P* < 0.001), and contribution ratio (*R*^2^ = 0.448, *P* < 0.001) of pre-anthesis DM under low yield levels (Figure 6), which suggests that under low yield levels improvements in the translocation amount and ratio of pre-anthesis DM are conducive to increased yield. However, in contrast, under high yield levels, the translocation amount, translocation ratio, and contribution ratio of pre-anthesis DM showed curves of a second-order equation with yield, with optimal values for higher yield of 3000–4500 kg ha^-1^, 25–35, and 30–50%, respectively.

**FIGURE 7:**
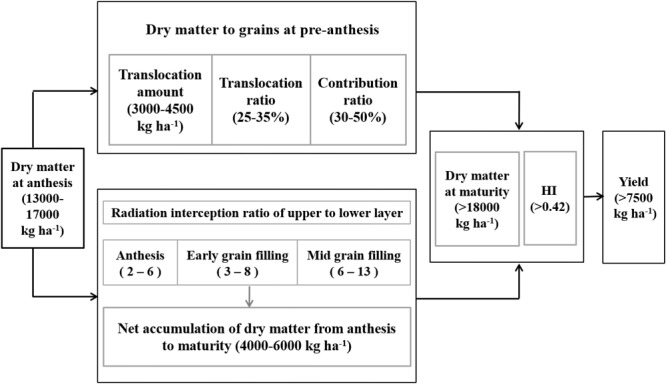
Growth indicators of increased yield at a yield level of >7500 kg ha^-1^ in winter wheat.

In summary, as shown in Figure 7, we propose physiological indicators of increased yield in the Huang-Huai Plain. First is an aboveground DM of 13,000–17,000 kg ha^-1^ at anthesis, providing the basis of transportation material and thereby supporting the canopy structure. Second, as mentioned above, is the translocation of pre-anthesis DM within an optimal range of 3000–4500 kg ha^-1^ under a translocation ratio of 25–35% and contribution ratio of 30–50%. Third, is an increase in DM from anthesis to maturity of 4000–6000 kg ha^-1^, with an RI ratio of upper to lower layers of 2–6, 3–8, and 6–13 at anthesis, early, and mid-grain filling, respectively. Lastly, is the optimal balance between aboveground DM at maturity (>18,000 kg ha^-1^) and HI (>0.42), which promotes yield levels of >7500 kg ha^-1^.

## Discussion

### Effect of Cultivation Strategy on DM Production, HI, and Yield

Water and nitrogen are the main limiting factors affecting crop growth (Li et al., 2009). Irrigation increases the availability of nitrogen, while nitrogen application promotes water utilization during wheat production, thereby contributing to plant growth by improving green leaf area, photosynthesis intensity, DM accumulation, and yield (Seiffert et al., 1995; Qiu et al., 2008). However, excessive irrigation and nitrogen fertilization lead to excessive vegetative growth and an increase in DM in stem and leaf organs at maturity, thereby reducing the HI (Zhao and Yu, 2006; Zhang and Yu, 2008). Previous studies suggest that irrigation at jointing plus anthesis, and a nitrogen application rate of 240–270 kg ha^-1^ are appropriate for achieving high yield (Zhang et al., 2009, 2011). Meanwhile, Wang C.Y. et al. (2014) found that irrigation at jointing plus booting and 240 kg N ha^-1^ resulted in the highest yield. In our study, DM, HI, and yield tended to increase with increasing irrigation and nitrogen application; however, a high N application rate of 300 kg N ha^-1^ under W2 caused reductions in DM and yield. Cultivation management patterns involve the integration of various cultivation techniques including tillage, fertilization, irrigation, and seeding (Chen et al., 2014; Wang Y.H. et al., 2014). Chen et al. (2014) found that optimized cultivation management significantly improved the total DM, HI, and yield compared to traditional farming practices. Wang Y.C. et al. (2015) further revealed that HH treatment could improve the DM_AM_ and HI compared to SH and FP. Our results are consistent with these findings, whereby HH, SH, and OFP treatment resulted in a higher DM_AM_ than FP, thereby increasing the HI.

Yield is determined by DM production, yield components, and HI, all of which show wide variation in different wheat production regions under diverse climate and soil conditions and between different cultivars (Unkovich et al., 2010; Terrile et al., 2017). Zhang et al. (2012) reported that increased yield could together with increasing HI without increase in DM, suggesting that yield could be improved via increased HI based on the same DM. In our study, yield, GN, DM, and HI showed wide distribution ranges, and the highest GN, DM, and HI were not always matched with the maximum yield in different locations (Tables 3–5 and Figure 2). With Yumai 49–198, a higher GN, DM_AM_, and yield but lower HI were observed in Shangshui, compared to Kaifeng and Wenxian. Meanwhile, with Aikang 58, smaller differences were observed between Shangshui and Wenxian. On the whole, however, the two cultivars showed consistency in terms of the relationship between yield, GN, DM, and HI. That is, in both cultivars at all three locations, increases in GN formed a larger sink, promoting high DM to match the high HI, thereby resulting in an increase in yield.

### Contributions of DM and HI to Increased Yield

Recently, increases in wheat yield have been attributed to increases in HI (Foulkes et al., 2009; Aranjuelo et al., 2013). Moreover, since the green revolution and genetic improvements in breeding, HI values have continued to reach high levels (Wiesmeier et al., 2014; Fan et al., 2017). As a result, it has been suggested that further increases in yield are now dependent on increases in total DM, providing a more solid foundation for yield formation (Parry et al., 2010; Aranjuelo et al., 2013). However, in reality, increases in both HI and total DM are conducive to improvements in yield within a certain range of production. Moreover, the contributions of HI and total DM on yield differ between ecological regions and different productivity levels (Lynch et al., 2017). Kawano (1990) reported that in rice the total DM is more important under low yielding levels, while the HI has a more significant effect under high yielding levels. Moreover, Rose et al. (2017) studied winter wheat production in Central Europe and found that while both HI and total DM are important in increasing yield, HI is more important. In our study, both total DM and HI were important under low yield levels, while HI was more important under high yield levels. These findings may be due to the long vegetative period and suitable light and temperature conditions before anthesis, which result in high DM levels under high yield levels in the Huang-Huai Plain.

Total DM can be divided into pre- and post-anthesis DM, which shows differences in determining yield by affecting GN and TGW. In Mediterranean regions, wheat yield is largely dependent on pre-anthesis DM production because of the favorable rainfall and temperature conditions, with hot dry weather after anthesis having an adverse effect on yield formation (Papakosta and Gagianas, 1991; Ercoli et al., 2008). However, in other eco-climatic regions, post-anthesis DM production may be more important (Zhang et al., 2012). Net accumulation of post-anthesis DM is mainly stored in the grains and is therefore closely related to yield (Zhang et al., 2016). Our study confirmed the importance of post-anthesis DM on increasing yield under high yield levels, while pre-anthesis DM was found to play a more important role in yield under low yield levels.

### Effect of DM Production During Filling on HI

HI is significantly affected by various morphological and physiological traits including plant height and DM partitioning into the spikes (Sayre et al., 1997; Zhang et al., 2012). However, with breeding of dwarf genes into modern wheat and plant height reaching an optimal level, the effect on HI has become weaker (Zhang et al., 2012). Increased partitioning of photosynthates to grains could improve HI via adequate reductions in the structure of the stem, rachis, glume, and palea (Palta et al., 1994; Foulkes et al., 2011). Thus, increases in HI could improve yield under the same DM. In this study, HI tended to show a parabolic relationship with total DM. Specifically, HI was positively correlated with total DM under low yield levels, but negatively under high yield levels. These findings suggest that higher yield is achieved via a balance between total DM and HI, while excessive DM production breaks this balance, subsequently reducing HI and decreasing yield. Further studies suggest that improved post-anthesis remobilization of water-soluble carbohydrates in the stem, leaf, and sheath to the grains also increase HI (Yang et al., 2000; Foulkes et al., 2007). Net accumulation of DM during filling also plays a very important role in ensuring a high sink (Bustos et al., 2013; Zhang et al., 2016). A larger sink provides a sufficient structural foundation for storing assimilates, and therefore, improving HI (Zhang et al., 2012). Our results are consistent with this, with a significant positive correlation between GN and HI, which requires more photosynthates during filling to meet the demands of the sink. Compared to total DM, net accumulation of DM during filling was important in terms of increased HI under low yield levels, but significantly alleviated the negative correlation with HI under high yield levels, thereby contributing to reaching a new balance between high HI and total DM in terms of increased yield. Overall, therefore, maintaining high net accumulation of DM during filling is beneficial in terms of achieving high HI under both high and low yield levels.

### Canopy Architecture Characteristics of RI and LAI During Grain Filling

Effective canopy architecture provides a suitable leaf layout and space structure for adaptation to different light radiation environments (Zhang et al., 2003; Dong et al., 2015). Optimal canopy architecture delays leaf senescence by adjusting the temperature and humidity to allow interception of more light radiation. Previous research suggests that interception of light radiation is determined by the leaf area and plant type (Ceotto et al., 2013; Du et al., 2015). In this study, we examined LAP and corresponding RIP, revealing an increase in RIP with increasing LAP. In line with this, some studies suggest that DM production relies on the ability of the canopy to intercept light radiation via the function of green leaves and conversion of solar energy into DM (Miralles and Slafer, 1997; Reynolds et al., 2005; Schierenbeck et al., 2016). In our study, both LAP and RIP showed close correlations with DM production, where a higher LAP and RIP after anthesis contributed to increases in net DM accumulation after anthesis. A longer green leaf duration and higher radiation interception after anthesis are therefore particularly important in terms of DM and yield.

Previous studies also suggest that LAI accurately reflects the radiation interception capacity and canopy architecture (Plenet et al., 2000; Li et al., 2010). An optimal crop canopy is able to effectively intercept and use solar radiation, thereby promoting photosynthesis and DM production (Li et al., 2010). The vertical distribution of leaves therefore has a direct effect on the interception and distribution of radiation within the canopy. Li et al. (2010) reported that the leaf area density within the upper and middle layers of the canopy increases with plant growth, while that of the lower layer decreases. In our study, upper- and lower-layer LAI under different total DM levels was positively correlated with yield. Compared to low yield levels, a higher lower-layer leaf area was more important in terms of yield under high yield levels. This was possibly due to the effect of maintaining more lower-layer leaves and delaying leaf senescence to intercept more radiation, with more nutriment being produced in lower layers and preferentially transferred to the root organs. Canopy architecture and leaf area both have effects on radiation interception (Du et al., 2015). In this study, the relationship between yield and the ratio of upper- to lower-layer RI followed curves of a second-order equation at different stages. The optimal ratios of upper- to lower-layer RI at anthesis, early, and mid-filling were, respectively, within the ranges of 2–6, 3–8, and 6–13 under high total DM at anthesis of >13,000 kg ha^-1^, and 6–10, 8–13, and 12–25, respectively, under low total DM at anthesis of <13,000 kg ha^-1^. Values above and below these optimal ratio ranges reduce radiation interception, leading to less effective use of radiation. In addition, under high total DM at anthesis, the optimal ratio was lower than that under low total DM, suggesting that a higher RI ratio in lower layers is beneficial to yield due to increased interception and use of radiation in lower layers, and therefore, resulting in increased production of photosynthetic assimilates.

### Characteristics of DM Production and Translocation

In general, grain yield is the result of DM assimilation and translocation of mobilized nutrients from vegetative organs (Sadras and Lawson, 2011). Previous studies suggest that increased allocation of assimilates to reproductive organs helps improve the sink and yield potential (Richards, 2000; Zhang et al., 2008). Fischer (1981) suggested that high DM production at post-anthesis tends to promote storage of assimilates in grains compared to stems and leaves, thereby increasing HI and yield. In our study, under low yield levels, accelerated translocation of assimilates from pre-anthesis vegetative organs to grains and a higher contribution ratio of translocation to grains helped increase yield. Under high yield levels, the relationship between the translocation and contribution of pre-anthesis assimilates and yield followed a second-order curve, with an optimal translocation amount of 3000–4500 kg ha^-1^, translocation ratio of 25–35%, and contribution ratio of 30–50%. These findings suggest that maintaining the balance between DM production at pre- and post-anthesis, to ensure higher post-anthesis DM productivity, contributes to increases in HI, and therefore, higher yield.

The physiological indicators of increased yield differ between different regions and productivity levels (Papakosta and Gagianas, 1991; Rose et al., 2017). In the Mediterranean regions, yield is largely dependent on the translocation of pre-anthesis assimilates, with total DM at maturity generally <15,000 kg ha^-1^ (Ercoli et al., 2008). Meanwhile, in the high rainfall zones of southern Australia, yield is correlated with total post-anthesis net DM accumulation and translocation of pre-anthesis assimilates, with the total DM at maturity generally being <18,000 kg ha^-1^ (Zhang et al., 2012). In this study, net DM accumulation ranged between 4000 and 6000 kg ha^-1^ during filling, resulting in a HI value of >0.42 and DM at maturity value of >18,000 kg ha^-1^, thereby reaching higher yield. This study was conducted in multiple sites across several years, providing an important basis for future breeding programs and the production of wheat in the Huang-Huai Plain. Further studies are now required to determine the appropriate field management aimed at promoting net accumulation of DM during filling.

## Conclusion

In the Huang-Huai Plain, increased yield under low yield levels was attributed to increases in both total DM and HI, while under high yield levels increasing yield was largely dependent on further improvement in HI. Under high yield levels, high net DM accumulation during filling reached 4000–6000 kg ha^-1^ via increases in LAP and RIP, thereby resulting in a high HI. The ratios of upper- to lower-layer RI showed curves of a second-order equation with yield after anthesis. Meanwhile, under high total DM levels, higher lower-layer LAI allowed interception of more radiation, with a decrease in the interception ratio of upper to lower layers, thereby increasing post-anthesis DM production and yield. Moreover, for the contribution of pre-anthesis DM to grains, accelerating DM mobilization from vegetative organs to grains was beneficial to yield. In this region, the following growth indicators were subsequently proposed to achieve higher yield: DM at anthesis of 13,000–17,000 kg ha^-1^, net DM accumulation after anthesis of 4000–6000 kg ha^-1^, DM at maturity of >18,000 kg ha^-1^, and HI of >0.42. Under the climatic conditions of the three experimental locations, cultivars Yumai 49–198 and Aikang 58 showed the same pathway to increase yield, i.e., an increase in GN to form a larger sink and high net accumulation of DM during filling increases HI, thereby contributing to increases in yield. Hence, N applied as basal fertilizer and irrigation after sowing should be appropriately reduced to promote root growth downward and breed strong seedling; N topdressing (50–60%) and irrigation should be applied in jointing for optimizing canopy structure and improving sink. Meanwhile, supplementary irrigation at anthesis contributes to improving plant activity, delaying leaf senescence, and producing more net DM, and therefore, increasing HI and improving yield.

## Author Contributions

WF, YuZ, and TG designed the experiments. JD, YW, YiZ, and XR carried out the experiments. JD and WF analyzed the experimental results and wrote the manuscript. YS and LH assisted in writing and editing the manuscript.

## Conflict of Interest Statement

The authors declare that the research was conducted in the absence of any commercial or financial relationships that could be construed as a potential conflict of interest.

## References

[B1] Aranjuelo I. Sanzsaez A. Jauregui I. Irigoyen J. J. Araus J. L. Sanchezdiaz M. (2013). Harvest index, a parameter conditioning responsiveness of wheat plants to elevated CO2. *J. Exp. Bot.* 64 1879–1892. 10.1093/jxb/ert081 23564953 PMC3638836

[B2] Austin R. B. (1980). “Physiological limitations to cereals yields and ways of reducing them by breeding,” in *Opportunities for Increasing Crop Yields* eds Hurd R. G. Biscoe P. V. Dennis C. (London: Association of Applied Biology/Pitman) 3–9.

[B3] Bustos D. V. Hasan A. K. Reynolds M. P. Calderini D. F. (2013). Combining high grain number and weight through a DH-population to improve grain yield potential of wheat in high-yielding environments. *Field Crops Res.* 145 106–115. 10.1016/j.fcr.2013.01.015

[B4] Ceotto E. Candilo M. D. Castelli F. Badeck F. W. Rizza F. Soave C. (2013). Comparing solar radiation interception and use efficiency for the energy crops giant reed (*Arundo donax* L.) and sweet sorghum (*Sorghum bicolor* L. Moench). *Field Crops Res.* 149 159–166. 10.1016/j.fcr.2013.05.002

[B5] Chen X. P. Cui Z. L. Fan M. S. Vitousek P. Zhao M. Ma W. Q. (2014). Producing more grain with lower environmental costs. *Nature* 514 486–489. 10.1038/nature13609 25186728

[B6] Dong C. Shao L. Z. Liu G. H. Wang M. J. Liu H. Xie B. Z. (2015). Photosynthetic characteristics, antioxidant capacity and biomass yield of wheat exposed to intermittent light irradiation with millisecond-scale periods. *J. Plant Physiol.* 184 28–36. 10.1016/j.jplph.2015.06.012 26210319

[B7] Du X. B. Chen B. L. Shen T. Y. Zhang Y. X. Zhou Z. G. (2015). Effect of cropping system on radiation use efficiency in double-cropped wheat-cotton. *Field Crops Res.* 170 21–31. 10.1016/j.fcr.2014.09.013

[B8] Ercoli L. Lulli L. Mariotti M. Masoni A. Arduini I. (2008). Post-anthesis dry matter and nitrogen dynamics in durum wheat as affected by nitrogen supply and soil water availability. *Eur. J. Agron.* 28 138–147. 10.1016/j.eja.2007.06.002

[B9] Evans L. T. Fischer R. A. (1999). Yield potential: its definition, measurement, and significance. *Crop Sci.* 39 1544–1551. 10.2135/cropsci1999.3961544x

[B10] Fan J. Mcconkey B. Janzen H. Townley-Smith L. Wang H. (2017). Harvest index-yield relationship for estimating crop residue in cold continental climates. *Field Crops Res.* 204 153–157. 10.1016/j.fcr.2017.01.014

[B11] Fischer R. A. (1981). Optimizing the use of water and nitrogen through breeding of crops. *Plant Soil* 58 249–278. 10.1007/BF02180056

[B12] Fischer R. A. (1985). Number of kernels in wheat crops and the influence of solar radiation and temperature. *J. Agric. Sci.* 105 447–461. 10.1017/S0021859600056495

[B13] Foulkes M. Sylvester-Bradley R. Weightman R. Snape J. (2007). Identifying physiological traits associated with improved drought resistance in winter wheat. *Field Crops Res.* 103 11–24. 10.1016/j.fcr.2007.04.007

[B14] Foulkes M. J. Reynolds M. P. Sylvester-Bradley R. (2009). “Genetic improvement of grain crops: yield potential,” in *Crop Physiology: Applications for Genetic Improvement and Agronomy* eds Sadras V. O. Calderini D. F. (Burlinggton, NJ: Elsevier) 355–385. 10.1016/B978-0-12-374431-9.00015-3

[B15] Foulkes M. J. Slafer G. A. Davies W. J. Berry P. M. Sylvester-Bradley R. Martre P. (2011). Raising yield potential of wheat. III. Optimizing partitioning to grain while maintaining lodging resistance. *J. Exp. Bot.* 62 469–486. 10.1093/jxb/erq300 20952627

[B16] Kawano K. (1990). Harvest index and evolution of major food crop cultivars in the tropics. *Euphytica* 46 195–202. 10.1007/BF00027218

[B17] Li S. X. Wang Z. H. Malhi S. Li S. Q. Gao Y. J. Tian X. H. (2009). Nutrient and water management effects on crop production, and nutrient and water use efficiency in dryland areas of China. *Adv. Agron.* 102 223–265. 10.1016/S0065-2113(09)01007-4 29886197

[B18] Li Y.-d. Tang L. Zhang Y.-p. Zhu X.-c Cao W.-x. Zhu Y. (2010). Relationship of PAR interception of canopy to leaf area and yield in rice. *Sci. Agric. Sin.* 43 3296–3305. 10.3864/j.issn.0578-1752.2010.16.004

[B19] Lynch J. P. Doyle D. McAuley S. McHardy F. Danneels Q. Black L. C. (2017). The impact of variation in grain number and individual grain weight on winter wheat yield in the high yield potential environment of Ireland. *Eur. J. Agron.* 87 40–49. 10.1016/j.eja.2017.05.001

[B20] Merchukovnat L. Fahima T. Krugman T. Saranga Y. (2016). Ancestral QTL alleles from wild emmer wheat improve grain yield, biomass and photosynthesis across environments in modern wheat. *Plant Sci.* 251 23–34. 10.1016/j.plantsci.2016.05.003 27593460

[B21] Miralles D. J. Slafer G. A. (1997). Radiation interception and radiation use efficiency of near-isogenic wheat lines with different height. *Euphytica* 97 201–208. 10.1023/A:1003061706059

[B22] Nass H. G. (1980). Harvest index as a selection criterion for grain yield in two spring wheat crosses grown at two population densities. *Can. J. Plant Sci.* 60 1141–1146. 10.4141/cjps80-166

[B23] Palta J. A. Kobata T. Turner N. C. Fillery I. R. (1994). Remobilization of carbon and nitrogen in wheat as influenced by postanthesis water deficits. *Crop Sci.* 34 118–124. 10.2135/cropsci1994.0011183X003400010021x

[B24] Papakosta D. K. Gagianas A. A. (1991). Nitrogen and dry matter accumulation, remobilization, and losses for Mediterranean wheat during grain filling. *Agron. J.* 83 864–870. 10.2134/agronj1991.00021962008300050018x

[B25] Parry M. A. J. Hawkesford M. J. (2010). Food security: increasing yield and improving resource use efficiency. *Proc. Nutr. Soc.* 69 592–600. 10.1017/S0029665110003836 20860858

[B26] Parry M. A. J. Reynolds M. P. Salvucci M. E. Raines C. A. Andralojc P. J. Zhu X. (2010). Raising yield potential of wheat. II. Increasing photosynthetic capacity and efficiency. *J. Exp. Bot.* 62 453–467. 10.1093/jxb/erq304 21030385

[B27] Plenet D. Etchebest S. Mollier A. Pellerin S. (2000). Growth analysis of maize field crops under phosphorus deficiency. *Plant Soil* 223 119–132. 10.1023/A:1004877111238 29754325

[B28] Qin X. Zhang F. Liu C. Yu H. Cao B. Tian S. (2015). Wheat yield improvements in China: Past trends and future directions. *Field Crops Res.* 177 117–124. 10.1016/j.fcr.2015.03.013

[B29] Qin X. L. Weiner J. Qi L. Xiong Y. C. Li F. M. (2013). Allometric analysis of the effects of density on reproductive allocation and harvest index in 6 varieties of wheat (Triticum). *Field Crops Res.* 144 162–166. 10.1016/j.fcr.2012.12.011

[B30] Qiu G. Y. Wang L. M. He X. H. Zhang X. Y. Chen S. Y. Chen J. (2008). Water use efficiency and evapotranspiration of winter wheat and its response to irrigation regime in the north China plain. *Agric. For. Meteor.* 148 1848–1859. 10.1016/j.agrformet.2008.06.010

[B31] Reynolds M. Pellegrineschi A. Skovmand B. (2005). Sink-limitation to yield and biomass: a summary of some investigations in spring wheat. *Ann. Appl. Biol.* 146 39–49. 10.1111/j.1744-7348.2005.03100.x

[B32] Richards R. A. (2000). Selectable traits to increase crop photosynthesis and yield of grain crops. *J. Exp. Bot.* 51 447–458. 10.1093/jexbot/51.suppl_1.447 10938853

[B33] Rose T. Nagler S. Kage H. (2017). Yield formation of Central-European winter wheat cultivars on a large scale perspective. *Eur. J. Agron.* 86 93–102. 10.1016/j.eja.2017.03.003

[B34] Ru Z. G. Feng S. W. Li G. (2015). High-yield potential and effective ways of wheat in Yellow & Huai River Valley facultative winter wheat region. *Sci. Agric. Sin.* 48 3388–3393. 10.3864/j.issn.0578-1752.2015.17.006

[B35] Sadras V. O. Lawson C. (2011). Genetic gain in yield and associated changes in phenotype, trait plasticity and competitive ability of South Australian wheat varieties released between 1958 and 2007. *Crop Pasture Sci.* 62 533–549. 10.1071/CP11060

[B36] Sayre K. D. Rajaram S. Fischer R. A. (1997). Yield potential progress in short bread wheats in Northwest Mexico. *Crop Sci.* 37 36–42. 10.2135/cropsci1997.0011183X003700010006x

[B37] Schierenbeck M. Fleitas M. C. Miralles D. J. Simón M. R. (2016). Does radiation interception or radiation use efficiency limit the growth of wheat inoculated with tan spot or leaf rust? *Field Crops Res.* 199 65–76. 10.1016/j.fcr.2016.09.017

[B38] Seiffert S. Kaselowsky J. Jungk A. Claassen N. (1995). Observed and calculated potassium uptake by maize as affected by soil water content and bulk density. *Agron. J.* 87 1070–1077. 10.2134/agronj1995.00021962008700060007x

[B39] Slafer G. A. Satore E. A. Andrade F. H. (1994). “Increases in grain yield in bread wheat from breeding and associated physiological changes,” in *Genetic Improvement of Field Crops* ed. Slafer G. A. (New York, NY: Marcel Dekker) 1–68.

[B40] Slafer G. A. Savin R. Sadras V. O. (2014). Coarse and fine regulation of wheat yield components in response to genotype and environment. *Field Crops Res.* 157 71–83. 10.1016/j.fcr.2013.12.004

[B41] Terrile I. I. Miralles D. J. González F. G. (2017). Fruiting efficiency in wheat (*Triticum aestivum* L): Trait response to different growing conditions and its relation to spike dry weight at anthesis and grain weight at harvest. *Field Crops Res.* 201 86–96. 10.1016/j.fcr.2016.09.026

[B42] Unkovich M. Baldock J. Forbes M. (2010). “Chapter 5 - variability in harvest index of grain crops and potential significance for carbon accounting: examples from Australian agriculture,” in *Advances in Agronomy* ed. Donald L. S. (Cambridge, MA: Academic Press) 173–219. 10.1016/S0065-2113(10)05005-4

[B43] Wang C. Y. Liu W. X. Li Q. X. Ma D. Y. Lu H. F. Feng W. (2014). Effects of different irrigation and nitrogen regimes on root growth and its correlation with above-ground plant parts in high-yielding wheat under field conditions. *Field Crops Res.* 2014 138–149. 10.1016/j.fcr.2014.04.011

[B44] Wang H. G. Li D. X. Li Y. M. Li R. Q. (2015). Yield components and population and individual characteristics of growth and development of winter wheat over 10000 kg ha-1 in Hebei province. *Sci. Agric. Sin.* 14 2718–2729. 10.3864/j.issn.0578-1752.2015.14.004

[B45] Wang Y. C. Li C. X. Dai X. L. Zhou X. Y. Zhang Y. Li H. Y. (2015). Effects of cultivation patterns on the radiation use and grain yield of winter wheat. *Chin. J. Appl. Ecol.* 26 2707–2713. 10.13287/j.1001-9332.20150630.013 26785552

[B46] Wang Y. H. Hu W. L. Zhang X. L. Li L. X. Kang G. Z. Feng W. (2014). Effects of cultivation patterns on winter wheat root growth parameters and grain yield. *Field Crops Res.* 156 208–218. 10.1016/j.fcr.2013.11.017

[B47] Wei Y. M. Zhang P. Guan E. Q. Zhang G. Q. Zhang Y. Q. Song Z. M. (2013). Advances in study of quality property improvement of winter wheat in China. *Sci. Agric. Sin.* 20 4189–4196. 10.3864/j.issn.0578-1752.2013.20.002

[B48] Wiesmeier M. Hübner R. Dechow R. Maier H. Spörlein P. Geuß U. (2014). Estimation of past and recent carbon input by crops into agricultural soils of southeast Germany. *Eur. J. Agron.* 61 10–23. 10.1016/j.eja.2014.08.001

[B49] Yang J. C. Zhang J. H. Huang Z. L. Zhu Q. S. Wang L. (2000). Remobilization of carbon reserves is improved by controlled soil-drying during grain filling of wheat. *Crop Sci.* 40 1645–1655. 10.2135/cropsci2000.4061645x

[B50] Yu Q. Y. Wu W. B. You L. Z. Zhu T. J. Vliet J. V. Verburg P. H. (2017). Assessing the harvested area gap in China. *Agric. Syst.* 153 212–220. 10.1016/j.agsy.2017.02.003

[B51] Zhang F. Q. Wang X. Y. Yu Z. W. Wang X. Z. Bai H. L. (2009). Characteristics of accumulation and distribution of Nitrogen and dry Matter in wheat at yield level of ten thousand kilograms per hectare. *Acta Agron. Sin.* 35 1086–1096. 10.3724/SP.J.1006.2009.01086

[B52] Zhang H. P. Turner N. C. Poole M. L. (2012). Increasing the harvest index of wheat in the high rainfall zones of southern Australia. *Field Crops Res.* 129 111–123. 10.1016/j.fcr.2012.02.002

[B53] Zhang S. R. Ma K. P. Chen L. Z. (2003). Response of photosynthetic plasticity of *Paeonia suffruticosa* to changed light environments. *Environ. Exp. Bot.* 49 121–133. 10.1016/S0098-8472(02)00063-1

[B54] Zhang X. Y. Chen S. Y. Sun H. Y. Pei D. Wang Y. M. (2008). Dry matter, harvest index, grain yield and water use efficiency as affected by water supply in winter wheat. *Irrigation Sci.* 27 1–10. 10.1007/s00271-008-0131-2

[B55] Zhang Y. Xu W. G. Wang H. W. Dong H. B. Qi X. L. Zhao M. Z. (2016). Progress in genetic improvement of grain yield and related physiological traits of Chinese wheat in Henan Province. *Field Crops Res.* 199 117–128. 10.1016/j.fcr.2016.09.022

[B56] Zhang Y. L. Yu Z. W. (2008). Effects of irrigation amount on nitrogen uptake, distribution, use, and grain yield and quality in wheat. *Acta Agron. Sin.* 34 870–878. 10.3724/SP.J.1006.2008.00870

[B57] Zhang Y. P. Zhang Y. H. Wang Z. M. Wang Z. J. (2011). Characteristics of canopy structure and contributions of non-leaf organs to yield in winter wheat under different irrigated conditions. *Field Crops Res.* 123 187–195. 10.1016/j.fcr.2011.04.014

[B58] Zhao J. Y. Yu Z. W. (2006). Effects of nitrogen fertilizer rate on uptake, distribution and utilization of nitrogen in winter wheat under high yielding cultivated condition. *Acta Agron. Sin.* 32 484–490. 10.3321/j.issn:0496-3490.2006.04.003

